# Supplementary material for: Predicting functional effects of ion channel variants using new phenotypic machine learning methods
Source: PLoS Comput Biol. 2023 Mar 6;19(3):e1010959. doi: 10.1371/journal.pcbi.1010959 (PMC10019634; doi:10.1371/journal.pcbi.1010959)
Supplement: S1 Fig — a: Clinical descriptions from medical records or literature are harmonized by extracting relevant concepts and mapping them to the standardized vocabulary of HPO terms [1]. b: Each term represents a node in a directed acyclic graph. Edges denote directional IS-A (parent-child) relationships. During propagation, for each term in the set of terms, each parent of a term is included in the set of terms until the root node (HP:0000001 All) is included. Thus, the underlying knowledge graph allows the model to understand that, e.g., HP:0001249 Intellectual disability IS-A HP:0012759 Neurodevelopmental abnormality. Nodes are colored by term, with gray nodes not being present in the set of terms. c: Given two sets of terms, their pairwise semantic similarity is calculated as described above (Methods). In this example, the most informative common ancestor (MICA) method is shown. The information content (IC) of this node is −log2(f) where f is the frequency of the term over all sets of terms. In Resnik’s measure, the IC of the MICA is the pairwise similarity between the two sets of terms. d: The pairwise phenotypic similarity for each pair of n observations (variants) in the training data set is represented by a n-by-n square matrix. This matrix is a Gram matrix (or kernel matrix) if it is positive semi-definite. Here, a simplified low-dimensional representation of the resulting phenotypic similarity feature space is shown. The bold line is the hyperplane of our support-vector machine classifier, with the dashed lines being the soft margin. (PDF) [file pcbi.1010959.s001.pdf]

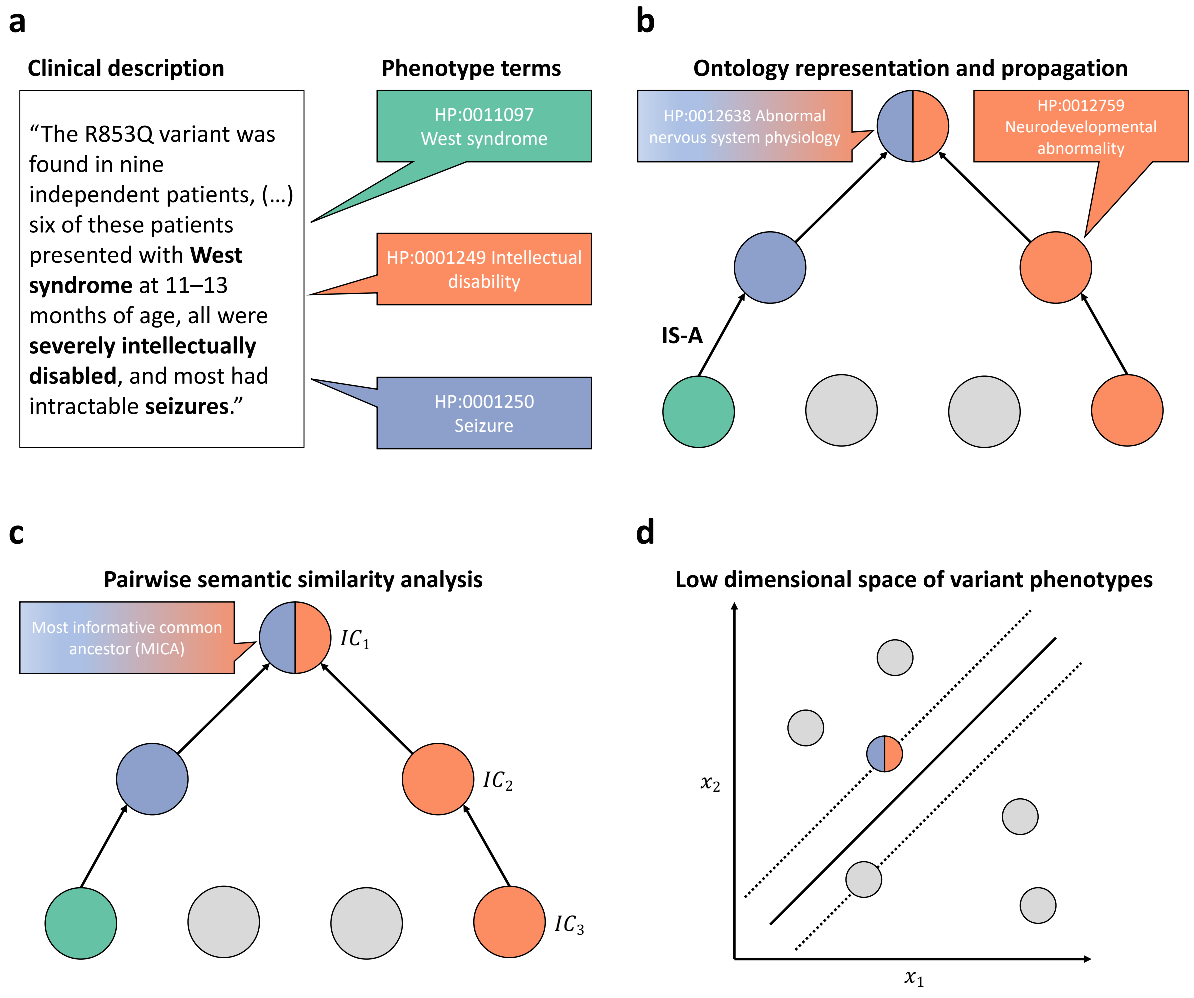

Figure S1. Human phenotype ontology (HPO) terms and phenotypic learning. *a*: Clinical descriptions from medical records or literature are harmonized by extracting relevant concepts and mapping them to the standardized vocabulary of HPO terms [1]. *b*: Each term represents a node in a directed acyclic graph. Edges denote directional IS-A (parent-child) relationships. During propagation, for each term in the set of terms, each parent of a term is included in the set of terms until the root node (HP:0000001 All) is included. Thus, the underlying knowledge graph allows the model to understand that, e.g., HP:0001249 Intellectual disability IS-A HP:0012759 Neurodevelopmental abnormality. Nodes are colored by term, with gray nodes not being present in the set of terms. *c*: Given two sets of terms, their pairwise semantic similarity is calculated as described above (Methods). In this example, the most informative common ancestor (MICA) method is shown. The information content (IC) of this node is $-\log_2(f)$ where f is the frequency of the term over all sets of terms. In Resnik's measure, the IC of the MICA is the pairwise similarity between the two sets of terms. *d*: The pairwise phenotypic similarity for each pair of n observations (variants) in the training data set is represented by a n-by-n square matrix. This matrix is a Gram matrix (or kernel matrix) if it is positive semi-definite. Here, a simplified low-dimensional representation of the resulting phenotypic similarity feature space is shown. The bold line is the hyperplane of our support-vector machine classifier, with the dashed lines being the soft margin.

Reference: [1] Wolff M, Johannesen KM, Hedrich UBS, Masnada S, Rubboli G, Gardella E, et al. Genetic and phenotypic heterogeneity suggest therapeutic implications in SCN2A-related disorders. Brain. 2017;140: 1316–1336. doi:10.1093/brain/awx054
