# Supplementary material for: Predicting functional effects of ion channel variants using new phenotypic machine learning methods
Source: PLoS Comput Biol. 2023 Mar 6;19(3):e1010959. doi: 10.1371/journal.pcbi.1010959 (PMC10019634; doi:10.1371/journal.pcbi.1010959)
Supplement: S2 Fig — a: Sequence- and structure-based pairwise similarity matrix. b: Task similarity matrix. c: Multi-task learning kernel matrix. d: Phenotypic similarity matrix. e: Multi-task multi-kernel learning matrix. (PDF) [file pcbi.1010959.s002.pdf]

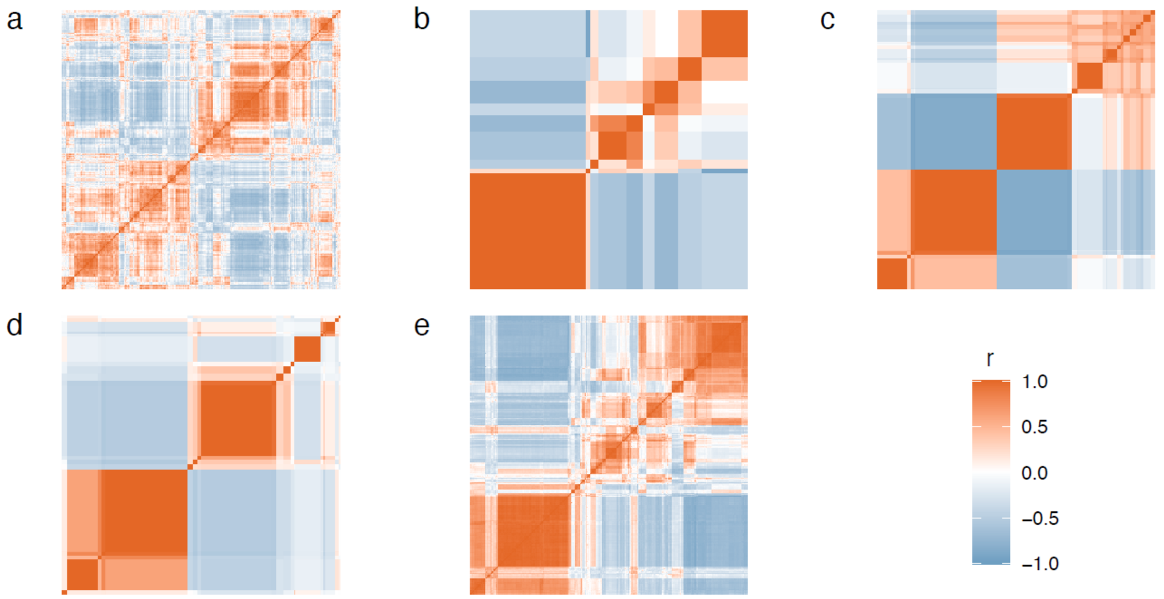

Figure S2. Kernel matrix visualization as correlation plots ordered by hierarchical clustering. *a*: Sequence- and structure-based pairwise similarity matrix. *b*: Task similarity matrix. *c*: Multi-task learning kernel matrix. *d*: Phenotypic similarity matrix. *e*: Multi-task multi-kernel learning matrix.
