# Supplementary material for: Predicting functional effects of ion channel variants using new phenotypic machine learning methods
Source: PLoS Comput Biol. 2023 Mar 6;19(3):e1010959. doi: 10.1371/journal.pcbi.1010959 (PMC10019634; doi:10.1371/journal.pcbi.1010959)
Supplement: S3 Fig — Node size and weight labels on the branches correspond to localized phenotypic similarity kernel weights. Weights were assumed to be uniform for SCN11A, as the sample size of the leaf subset was too small. Leaf-to-leaf distance on the dendrogram corresponds to the task similarity used for multi-task learning, with similar tasks clustered together. (PDF) [file pcbi.1010959.s003.pdf]

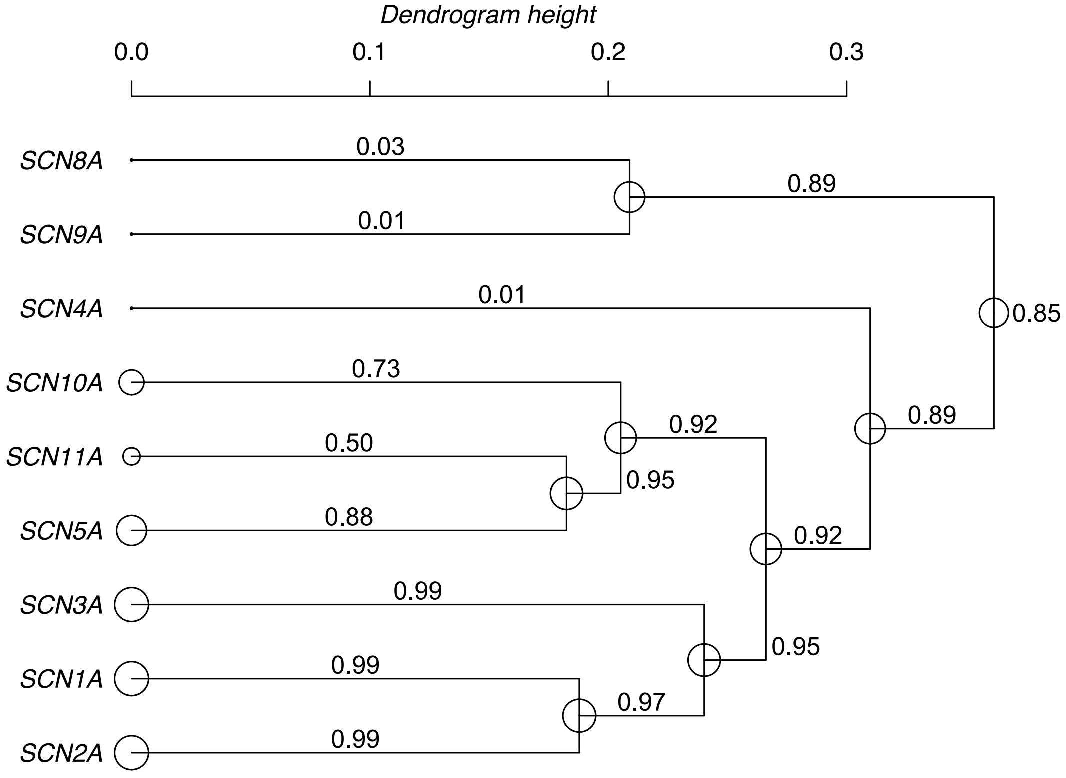

Figure S3. Dendrogram of hierarchical decomposition multi-task multi-kernel learning for voltage-gated sodium channels. Node size and weight labels on the branches correspond to localized phenotypic similarity kernel weights. Weights were assumed to be uniform for *SCN11A*, as the sample size of the leaf subset was too small. Leaf-to-leaf distance on the dendrogram corresponds to the task similarity used for multi-task learning, with similar tasks clustered together.
