# Supplementary material for: Predicting functional effects of ion channel variants using new phenotypic machine learning methods
Source: PLoS Comput Biol. 2023 Mar 6;19(3):e1010959. doi: 10.1371/journal.pcbi.1010959 (PMC10019634; doi:10.1371/journal.pcbi.1010959)
Supplement: S1 Table — Details on data curation and validation procedure are available in the Methods section. Primary disease corresponds to the core phenotype, while phenotype terms refers to the information provided to the MKL model. For the decision rule, exact matches are shown in bold. Abbreviations: BrS–Brugada syndrome; DEE–developmental and epileptic encephalopathy; DS–Dravet syndrome; ERS–Early repolarization syndrome; ESES—electrical status epilepticus during slow-wave sleep; GOF–gain-of-function; LOF–loss-of-function; LQT3 –long-QT syndrome 3; PMC–paramyotonia congenita; SeLIE–self-limited (familial) infantile epilepsy. (PDF) [file pcbi.1010959.s004.pdf]

Channel	Variant (p.)	Functional effect	Reference	Primary disease	Decision rule	Gradient boosting	Phenotype terms	MKL
SCN2A	Q1531K	GOF	[1]	See reference	NA	LOF 0.71	ORPHA:306	GOF 0.52
SCN2A	L1563V	GOF	[1]	See reference	NA	LOF 0.76	ORPHA:306	GOF 0.48
SCN2A	E1321K	GOF	[1]	See reference	NA	GOF 0.82	ORPHA:306	GOF 0.48
SCN2A	V1325I	GOF	[1]	See reference	NA	GOF 0.90	ORPHA:306, HP:0200134	GOF 0.46
SCN2A	D195G	LOF	[1]	See reference	NA	LOF 0.73	OMIM:619373	LOF 0.86
SCN2A	R853Q	LOF	[1]	See reference	*SCN4A* p.R672G: LOF, *SCN4A* p.R672H: LOF, *SCN5A* p.R811H: LOF	LOF 0.79	OMIM:619373, ORPHA:2382	LOF 0.89
SCN5A	V281A	LOF	[2]	BrS	NA	LOF 0.60	ORPHA:130	LOF 0.89
SCN5A	L1582P	LOF	[2]	BrS	NA	LOF 0.81	ORPHA:130	LOF 0.79
SCN5A	F543L	LOF	[2]	BrS	NA	GOF 0.52	ORPHA:130	LOF 0.73
SCN5A	K1419E	LOF	[2]	BrS	NA	LOF 0.60	ORPHA:130	LOF 0.87
SCN5A	L1412F	LOF	[3]	ERS	NA	LOF 0.66	HP:0031546	LOF 0.66
SCN5A	G452C	LOF	[3]	ERS	NA	LOF 0.68	HP:0031546	LOF 0.62
SCN1A	A1783V	LOF	[4]	DS	*SCN9A* p.A1746G: GOF, *SCN2A* p.A1773T: LOF	GOF 0.69	ORPHA:33069	LOF 0.88
SCN5A	R893C	LOF	[5]	BrS	***SCN1A* p.R946C: LOF**, *SCN1A* p.R946H: LOF, *SCN2A* p.R937C: LOF, *SCN2A* p.R937H: LOF	LOF 0.75	ORPHA:130	LOF 0.87
SCN5A	L846R	LOF	[5]	BrS	NA	GOF 0.51	ORPHA:130	LOF 0.78
SCN5A	G897E	LOF	[5]	BrS	NA	LOF 0.65	ORPHA:130	LOF 0.83
SCN5A	R878H	LOF	[5]	BrS	NA	LOF 0.78	ORPHA:130	LOF 0.86
SCN5A	G1743E	LOF	[5]	BrS	*SCN5A* p.G1743R: LOF	LOF 0.79	ORPHA:130	LOF 0.81
SCN4A	R1463H	GOF	[6]	PMC	NA	GOF 0.73	OMIM:168300	LOF 0.60
SCN5A	G1481V	GOF	[7]	LQT3	*SCN4A* p.G1306E: GOF, *SCN8A* p.G1745R: GOF	GOF 0.79	OMIM:603830	GOF 0.80
SCN5A	Q1491H	GOF	[7]	LQT3	NA	GOF 0.66	OMIM:603830	GOF 0.82
SCN5A	V1667D	LOF	[8]	BrS	NA	GOF 0.53	ORPHA:130	LOF 0.72
SCN2A	V911A	GOF	[9]	ESES, DEE	NA	LOF 0.60	HP:0031491, HP:0200134	GOF 0.55
SCN2A	K1933M	GOF	[9]	ESES, DEE	NA	LOF 0.60	HP:0031491, HP:0200134	GOF 0.52
SCN2A	S863F	LOF	[9]	SeLIE, ESES	NA	LOF 0.59	HP:0031491, ORPHA:306	LOF 0.84

SCN5A	R104W	LOF	[10]	BrS	*SCN4A* p.R104H: LOF, *SCN5A* p.R104Q: LOF	LOF 0.82	ORPHA:130	LOF 0.89
SCN5A	P1310L	LOF	[11]	BrS	NA	GOF 0.86	ORPHA:130	LOF 0.86
SCN9A	E44Q	GOF	[12]	Paroxysmal pain	NA	LOF 0.67	HP:0012531	GOF 0.81
SCN2A	R853Q	LOF	[13]	DEE	*SCN4A* p.R672G: LOF, *SCN4A* p.R672H: LOF, *SCN5A* p.R811H: LOF	LOF 0.79	HP:0200134	LOF 0.71
SCN5A	G1712S	LOF	[14]	BrS	***SCN10A* p.G1662S: GOF**	LOF 0.72	ORPHA:130	LOF 0.84

Table S1. Variants used for external validation. Details on data curation and validation procedure are available in the Methods section. Primary disease corresponds to the core phenotype, while phenotype terms refer to the information provided to the MKL model. For the decision rule, exact matches are shown in bold. Abbreviations: BrS – Brugada syndrome; DEE – developmental and epileptic encephalopathy; DS – Dravet syndrome; ERS – Early repolarization syndrome; ESES - electrical status epilepticus during slow-wave sleep; GOF – gain-of-function; LOF – loss-of-function; LQT3 – long-QT syndrome 3; PMC – paramyotonia congenita; SeLIE – self-limited (familial) infantile epilepsy.

References:

1. Berecki G, Howell KB, Heighway J, Olivier N, Rodda J, Overmars I, et al. Functional correlates of clinical phenotype and severity in recurrent SCN2A variants. Commun Biol. 2022;5: 515. doi:10.1038/s42003-022-03454-1

2. Mikhailova VB, Karpushev AV, Vavilova VD, Klimenko ES, Tulintseva T, Yudina YS, et al. Functional Analysis of SCN5A Genetic Variants Associated with Brugada Syndrome. Cardiology. 2022;147: 35–46. doi:10.1159/000519857

3. Zhang Z-H, Barajas-Martínez H, Xia H, Li B, Capra JA, Clatot J, et al. Distinct Features of Probands With Early Repolarization and Brugada Syndromes Carrying SCN5A Pathogenic Variants. J Am Coll Cardiol. 2021;78: 1603–1617. doi:10.1016/j.jacc.2021.08.024

4. Layer N, Sonnenberg L, Pardo González E, Benda J, Hedrich UBS, Lerche H, et al. Dravet Variant SCN1A A1783V Impairs Interneuron Firing Predominantly by Altered Channel Activation. Front Cell Neurosci. 2021;15: 754530. doi:10.3389/fncel.2021.754530

5. O’Neill MJ, Muhammad A, Li B, Wada Y, Hall L, Solus JF, et al. Dominant negative effects of SCN5A missense variants. Genet Med. 2022;24: 1238–1248. doi:10.1016/j.gim.2022.02.010

6. Brenes O, Barbieri R, Vásquez M, Vindas-Smith R, Roig J, Romero A, et al. Functional and Structural Characterization of ClC-1 and Nav1.4 Channels Resulting from CLCN1 and SCN4A Mutations Identified Alone and Coexisting in Myotonic Patients. Cells. 2021;10: 374. doi:10.3390/cells10020374

7. Plumereau Q, Theriault O, Pouliot V, Moreau A, Morel E, Fressart V, et al. Novel G1481V and Q1491H SCN5A Mutations Linked to Long QT Syndrome Destabilize the Nav1.5 Inactivation State. CJC Open. 2021;3: 256–266. doi:10.1016/j.cjco.2020.09.023

8. Monasky MM, Micaglio E, Ciconte G, Rivolta I, Borrelli V, Ghiroldi A, et al. Novel SCN5A p.Val1667Asp Missense Variant Segregation and Characterization in a Family with Severe

Brugada Syndrome and Multiple Sudden Deaths. Int J Mol Sci. 2021;22: 4700. doi:10.3390/ijms22094700

9. Miao P, Tang S, Ye J, Tang J, Wang J, Zheng C, et al. Differential Functional Changes of Nav1.2 Channel Causing SCN2A-Related Epilepsy and Status Epilepticus During Slow Sleep. Front Neurol. 2021;12: 653517. doi:10.3389/fneur.2021.653517

10. Doisne N, Grauso M, Mougenot N, Clergue M, Souil C, Coulombe A, et al. In vivo Dominant-Negative Effect of an SCN5A Brugada Syndrome Variant. Front Physiol. 2021;12: 661413. doi:10.3389/fphys.2021.661413

11. Balla C, Conte E, Selvatici R, Marsano RM, Gerbino A, Farnè M, et al. Functional Characterization of Two Novel Mutations in SCN5A Associated with Brugada Syndrome Identified in Italian Patients. Int J Mol Sci. 2021;22: 6513. doi:10.3390/ijms22126513

12. Takahashi K, Ohba T, Okamoto Y, Noguchi A, Okuda H, Kobayashi H, et al. E44Q mutation in NaV1.7 in a patient with infantile paroxysmal knee pain: electrophysiological analysis of voltage-dependent sodium current. Heliyon. 2021;7: e07396. doi:10.1016/j.heliyon.2021.e07396

13. Ganguly S, Thompson CH, George AL. Enhanced slow inactivation contributes to dysfunction of a recurrent SCN2A mutation associated with developmental and epileptic encephalopathy. J Physiol. 2021;599: 4375–4388. doi:10.1113/JP281834

14. Sanner K, Mueller-Leisse J, Zormpas C, Duncker D, Leffler A, Veltmann C. A Novel SCN5A Variant Causes Temperature-Sensitive Loss Of Function in a Family with Symptomatic Brugada Syndrome, Cardiac Conduction Disease, and Sick Sinus Syndrome. Cardiology. 2021;146: 754–762. doi:10.1159/000518210
