# Supplementary material for: Predicting functional effects of ion channel variants using new phenotypic machine learning methods
Source: PLoS Comput Biol. 2023 Mar 6;19(3):e1010959. doi: 10.1371/journal.pcbi.1010959 (PMC10019634; doi:10.1371/journal.pcbi.1010959)
Supplement: S2 Table — Raw and preprocessed feature tables are available as part of our ‘Availability of data and materials’ statement. (PDF) [file pcbi.1010959.s005.pdf]

Feature	Description	Data type	Reference
cid	relative variant position on the gene family multiple sequence alignment via MUSCLE with UpgmaMax clustering	numerical	[1]
gene	vector of task membership used for multi-task learning	categorical	–
aa_blosum62	amino acid substitution value according to the midrange Blocks Substitution Matrix (BLOSUM62)	numerical	[2]
aa_blomap_aa1_dim1	encoding of the physicochemical properties of the original amino acid using a Naïve Bayes classifier, first encoding dimension	numerical	[3]
aa_blomap_aa1_dim2	encoding of the physicochemical properties of the original amino acid using a Naïve Bayes classifier, second encoding dimension	numerical	[3]
aa_blomap_aa1_dim3	encoding of the physicochemical properties of the original amino acid using a Naïve Bayes classifier, third encoding dimension	numerical	[3]
aa_blomap_aa1_dim4	encoding of the physicochemical properties of the original amino acid using a Naïve Bayes classifier, fourth encoding dimension	numerical	[3]
aa_blomap_aa1_dim5	encoding of the physicochemical properties of the original amino acid using a Naïve Bayes classifier, fifth encoding dimension	numerical	[3]
aa_blomap_aa2_dim1	encoding of the physicochemical properties of the substituted amino acid using a Naïve Bayes classifier, first encoding dimension	numerical	[3]
aa_blomap_aa2_dim2	encoding of the physicochemical properties of the substituted amino acid using a Naïve Bayes classifier, second encoding dimension	numerical	[3]
aa_blomap_aa2_dim3	encoding of the physicochemical properties of the substituted amino acid using a Naïve Bayes classifier, third encoding dimension	numerical	[3]
aa_blomap_aa2_dim4	encoding of the physicochemical properties of the substituted amino acid using a Naïve Bayes classifier, fourth encoding dimension	numerical	[3]
aa_blomap_aa2_dim5	encoding of the physicochemical properties of the substituted amino acid using a Naïve Bayes classifier, fifth encoding dimension	numerical	[3]
aa_braun_aa1_E1	encoding of the physicochemical properties of the original amino acid using multidimensional scaling, first encoding dimension	numerical	[4]
aa_braun_aa1_E2	encoding of the physicochemical properties of the original amino acid using multidimensional scaling, second encoding dimension	numerical	[4]
aa_braun_aa1_E3	encoding of the physicochemical properties of the original amino acid using multidimensional scaling, third encoding dimension	numerical	[4]
aa_braun_aa1_E4	encoding of the physicochemical properties of the original amino acid using multidimensional scaling, fourth encoding dimension	numerical	[4]
aa_braun_aa1_E5	encoding of the physicochemical properties of the original amino acid using multidimensional scaling, fifth encoding dimension	numerical	[4]
aa_braun_aa2_E1	encoding of the physicochemical properties of the substituted amino acid using multidimensional scaling, first encoding dimension	numerical	[4]
aa_braun_aa2_E2	encoding of the physicochemical properties of the substituted amino acid using multidimensional scaling, second encoding dimension	numerical	[4]
aa_braun_aa2_E3	encoding of the physicochemical properties of the substituted amino acid using multidimensional scaling, third encoding dimension	numerical	[4]
aa_braun_aa2_E4	encoding of the physicochemical properties of the substituted amino acid using multidimensional scaling, fourth encoding dimension	numerical	[4]
aa_braun_aa2_E5	encoding of the physicochemical properties of the substituted amino acid using multidimensional scaling, fifth encoding dimension	numerical	[4]
aa_grantham	Grantham distance between the original and substituted amino acids, based on composition, polarity and residue volume	numerical	[5]
aa_hphob_pca1	change in hydrophobicity by amino acid substitution using 98 hydrophobicity scales and principal component analysis, first component	numerical	[6]

aa_hphob_pca2	change in hydrophobicity by amino acid substitution using 98 hydrophobicity scales and principal component analysis, first component	numerical	[6]
aa_hphob_pca3	change in hydrophobicity by amino acid substitution using 98 hydrophobicity scales and principal component analysis, first component	numerical	[6]
str_np_rsa	change in residue relative accessible surface area predicted by NetSurfP – 2.0	numerical	[7]
str_np_asa	change in residue accessible surface area predicted by NetSurfP – 2.0	numerical	[7]
str_paraz	amino acid conservation across gene paralogs (Parazscore)	numerical	[8]
str_iupred	energy estimation-based structural state prediction for ordered and disordered residues by IUPred2	numerical	[9]
str_anchor	energy estimation-based structural state prediction for disordered binding regions by ANCHOR2	numerical	[9]
str_asa	change in residue accessible surface area predicted by PROF via PredictProtein	numerical	[10]
str_rsa	change in residue relative accessible surface area predicted by PROF via PredictProtein	numerical	[10]
str_helix	prediction of topology for helical transmembrane proteins by PHDhtm_top via PredictProtein, network output for helix	numerical	[10]
str_loop	prediction of topology for helical transmembrane proteins by PHDhtm_top via PredictProtein, network output for loop	numerical	[10]
str_nors	prediction of protein disorder by NORSnet via PredictProtein	numerical	[10,11]
str_profbval	prediction of protein disorder by PROFbval via PredictProtein	numerical	[10,12]
str_ucon	prediction of protein disorder by Ucon via PredictProtein	numerical	[10,13]
str_isis	prediction of residue involvement in protein-protein interaction sites by ISIS	numerical	[14]
str_consurf	probabilistic positional evolutionary conservation estimates by ConSurf via PredictProtein	numerical	[10,15]
ecdf	relative variant position along sequence length (empirical distribution function)	numerical	–
str_dom1	position on protein topology via UniProt, binary encoding	numerical	[16]
str_dom2	position on protein topology via UniProt, binary encoding	numerical	[16]
str_dom3	position on protein topology via UniProt, binary encoding	numerical	[16]
str_dom4	position on protein topology via UniProt, binary encoding	numerical	[16]
str_dom5	position on protein topology via UniProt, binary encoding	numerical	[16]
str_np_q3_C	3-class secondary structure classification (SS3) by NetSurfP – 2.0 and DSSP	categorical	[7,17]
str_np_q3_E	3-class secondary structure classification (SS3) by NetSurfP – 2.0 and DSSP	categorical	[7,17]
str_np_q3_H	3-class secondary structure classification (SS3) by NetSurfP – 2.0 and DSSP	categorical	[7,17]
str_np_q8_B	8-class secondary structure classification (SS8) by NetSurfP – 2.0 and DSSP	categorical	[7,17]
str_np_q8_C	8-class secondary structure classification (SS8) by NetSurfP – 2.0 and DSSP	categorical	[7,17]
str_np_q8_E	8-class secondary structure classification (SS8) by NetSurfP – 2.0 and DSSP	categorical	[7,17]

str_np_q8_G	8-class secondary structure classification (SS8) by NetSurfP – 2.0 and DSSP	categorical	[7,17]
str_np_q8_H	8-class secondary structure classification (SS8) by NetSurfP – 2.0 and DSSP	categorical	[7,17]
str_np_q8_I	8-class secondary structure classification (SS8) by NetSurfP – 2.0 and DSSP	categorical	[7,17]
str_np_q8_S	8-class secondary structure classification (SS8) by NetSurfP – 2.0 and DSSP	categorical	[7,17]
str_np_q8_T	8-class secondary structure classification (SS8) by NetSurfP – 2.0 and DSSP	categorical	[7,17]
str_pbie_b	state of residue relative accessible surface area predicted by PROF via PredictProtein, buried	categorical	[10,12]
str_pbie_e	state of residue relative accessible surface area predicted by PROF via PredictProtein, exposed	categorical	[10,12]
str_pbie_i	state of residue relative accessible surface area predicted by PROF via PredictProtein, intermediate	categorical	[10,12]
str_prhl_H	maximum score model prediction of topology for helical transmembrane proteins by PHDhtm_top via PredictProtein, helical membrane	categorical	[10]
str_prhl_L	maximum score model prediction of topology for helical transmembrane proteins by PHDhtm_top via PredictProtein, no helical transmembrane	categorical	[10]
str_pito_i	predicted topology of transmembrane regions by PHDhtm_top via PredictProtein, loop inside	categorical	[10]
str_pito_o	predicted topology of transmembrane regions by PHDhtm_top via PredictProtein, loop outside	categorical	[10]
str_pito_T	predicted topology of transmembrane regions by PHDhtm_top via PredictProtein, transmembrane	categorical	[10]

Table S2. List and description of sequence- and structure-based features, sorted by column vectors in the training data set. Raw and preprocessed feature tables are available as part of our 'Availability of data and materials' statement.

References:

1. Edgar RC. MUSCLE: a multiple sequence alignment method with reduced time and space complexity. BMC Bioinformatics. 2004;5: 113. doi:10.1186/1471-2105-5-113

2. Henikoff S, Henikoff JG. Amino acid substitution matrices from protein blocks. Proc Natl Acad Sci U S A. 1992;89: 10915–10919. doi:10.1073/pnas.89.22.10915

3. Maetschke SR, Towsey M, Boden MB. BLOMAP: An encoding of amino acids which improves signal peptide cleavage site prediction. Series on Advances in Bioinformatics and Computational Biology. Imperial College Press; 2005. pp. 141–150. doi:10.1142/9781860947322_0014

4. Venkatarajan M, Braun W. New quantitative descriptors of amino acids based on multidimensional scaling of a large number of physical-chemical properties. Journal of Molecular Modeling. 2001;7: 445–453. doi:10.1007/s00894-001-0058-5

5. Grantham R. Amino acid difference formula to help explain protein evolution. Science. 1974;185: 862–864. doi:10.1126/science.185.4154.862

6. Simm S, Einloft J, Mirus O, Schleiff E. 50 years of amino acid hydrophobicity scales: revisiting the capacity for peptide classification. Biol Res. 2016;49: 31. doi:10.1186/s40659-016-0092-5

7. Klausen MS, Jespersen MC, Nielsen H, Jensen KK, Jurtz VI, Sønderby CK, et al. NetSurfP-2.0: Improved prediction of protein structural features by integrated deep learning. Proteins. 2019;87: 520–527. doi:10.1002/prot.25674

8. Lal D, May P, Perez-Palma E, Samocha KE, Kosmicki JA, Robinson EB, et al. Gene family information facilitates variant interpretation and identification of disease-associated genes in neurodevelopmental disorders. Genome Med. 2020;12: 28. doi:10.1186/s13073-020-00725-6

9. Mészáros B, Erdos G, Dosztányi Z. IUPred2A: context-dependent prediction of protein disorder as a function of redox state and protein binding. Nucleic Acids Res. 2018;46: W329–W337. doi:10.1093/nar/gky384

10. Bernhofer M, Dallago C, Karl T, Satagopam V, Heinzinger M, Littmann M, et al. PredictProtein - Predicting Protein Structure and Function for 29 Years. Nucleic Acids Res. 2021;49: W535–W540. doi:10.1093/nar/gkab354

11. Schlessinger A, Punta M, Rost B. Natively unstructured regions in proteins identified from contact predictions. Bioinformatics. 2007;23: 2376–2384. doi:10.1093/bioinformatics/btm349

12. Schlessinger A, Yachdav G, Rost B. PROFbval: predict flexible and rigid residues in proteins. Bioinformatics. 2006;22: 891–893. doi:10.1093/bioinformatics/btl032

13. Schlessinger A, Liu J, Rost B. Natively unstructured loops differ from other loops. PLoS Comput Biol. 2007;3: e140. doi:10.1371/journal.pcbi.0030140

14. Ofran Y, Rost B. ISIS: interaction sites identified from sequence. Bioinformatics. 2007;23: e13-16. doi:10.1093/bioinformatics/btl303

15. Ashkenazy H, Abadi S, Martz E, Chay O, Mayrose I, Pupko T, et al. ConSurf 2016: an improved methodology to estimate and visualize evolutionary conservation in macromolecules. Nucleic Acids Res. 2016;44: W344-350. doi:10.1093/nar/gkw408

16. Apweiler R, Bairoch A, Wu CH, Barker WC, Boeckmann B, Ferro S, et al. UniProt: the Universal Protein knowledgebase. Nucleic Acids Res. 2004;32: D115-119. doi:10.1093/nar/gkh131

17. Kabsch W, Sander C. Dictionary of protein secondary structure: pattern recognition of hydrogen-bonded and geometrical features. Biopolymers. 1983;22: 2577–2637. doi:10.1002/bip.360221211
